# Spinal Cord Infarction Thrombolysed at Seven Hours: A Case Report and Review of Literature

**DOI:** 10.7759/cureus.55983

**Published:** 2024-03-11

**Authors:** Saket N Chandak, Nitin Chandak, Dinesh Kabra, Neeraj Baheti

**Affiliations:** 1 Neurology, Jawaharlal Nehru Medical College, Datta Meghe Institute of Medical Sciences, Nagpur, IND; 2 Neurology, Dr. G. M. Taori Central India Institute of Medical Sciences, Nagpur, IND

**Keywords:** outcome, paraplegia, thrombolysis, infarction, spinal cord

## Abstract

We report a case of acute spinal cord infarction treated with intravenous (IV) thrombolysis at seven hours from symptom onset. Nineteen previously thrombolysed cases are reviewed. The patient underwent a clinical assessment, followed by an MRI of the spine. He was thrombolysed with a recombinant tissue plasminogen activator. Neurological severity was assessed at presentation and 24 hours using the National Institute of Health Stroke Scale (NIHSS), and disability at three months was evaluated using a modified Rankin scale (mRS). A middle-aged man presented with acute-onset paraplegia (NIHSS 9). MRI with T2-weighted sagittal, axial, and diffusion-weighted images showed hyperintensity from D10 to LI vertebral levels. He was thrombolysed at 428 minutes, leading to mild clinical improvement at 24 hours (NIHSS 7). At three months, he could walk with support (mRS 3). Nineteen cases of acute spinal cord infarction treated with IV thrombolysis have been reported. Clinical outcome at three months is available for 16 patients: seven (44%) had a good outcome (mRS 0-2); this is the first reported case of spinal cord infarction treated with thrombolysis at seven hours. Clinical trials to confirm the efficacy and safety of thrombolysis in spinal cord infarcts are needed.

## Introduction

Spinal cord infarctions are uncommon and constitute around 1.2% of stroke patients. Spinal cord infarctions may be misdiagnosed due to low clinical suspicion. Fourteen to 16 percent of patients referred to a referral center with transverse myelitis had ischemic myelopathy; these infarcts are either spontaneous or periprocedural [1,2]. Currently, there are no guidelines for the treatment of spinal cord injuries. In a retrospective evaluation of 57 spinal cord infarction patients with a median follow-up of 2.1 years, only 21 (38%) were ambulatory with or without walking aid, and 12 (21%) died [3]. The severity of motor deficits at presentation and advanced age are poor prognostic factors [4]. We present a case of acute paraplegia due to a spinal cord infarction treated with intravenous (IV) thrombolysis at seven hours.

## Case presentation

**Figure 1:**
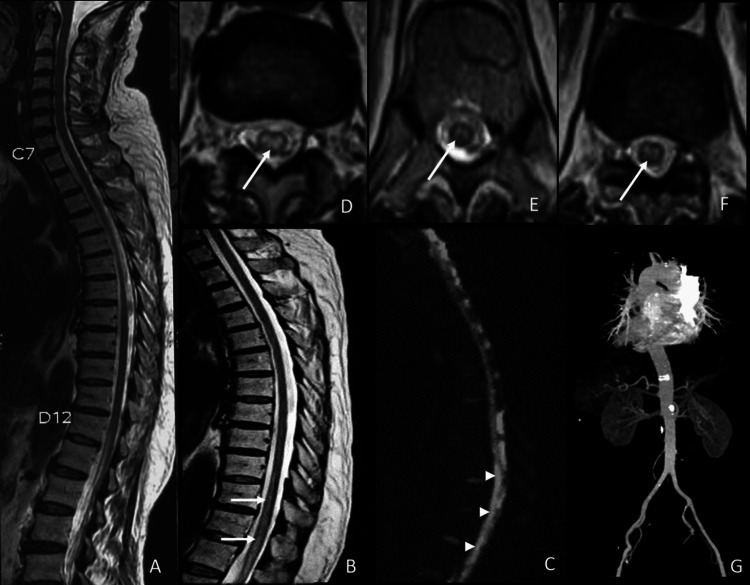
MRI of the spinal cord Normal T2-weighted sagittal MR image (A) at three hours. At five hours, sagittal T2-weighted (B) and diffusion-weighted (C) MR images showed linear pencil-like hyperintensity in the anterior two-thirds of the chord from D10 to L1 level (white-arrow). Axial T2-weighted MR images (D-F) showing hyperintensity in the corresponding regions (arrowheads). CT angiography (G) shows few calcifications. MRI: magnetic resonance imaging

A gentleman in his late 50s presented with acute-onset weakness in his legs, tingling and numbness below the waist, and difficulty passing urine. He had mild lower back pain. He had hypertension, but he did not take regular antihypertensive therapy, and he did not consume alcohol or tobacco. Blood pressure was 130/80 mm Hg. He was conscious, alert, and oriented. The cranial nerve examination was normal. Motor strength was normal in both upper extremities. The lower extremities on the right had grade 0 power; the lower extremities on the left had grade 1 power. Touch and pinprick sensations were impaired below the inguinal region bilaterally. Joint position sense was impaired in both great toes. Stroke severity on the National Institute of Health Stroke Scale (NIHSS) was 9. The plantar reflex was extended bilaterally. Screening T2 weighted (T2W) magnetic resonance imaging (MRI) of the spine done elsewhere was normal. Repeat MRI of the dorsal spine at five hours from symptom onset showed T2-weighted and DWI hyperintensity in the anterior 2/3rd extending from D10 to L1, suggestive of acute spinal infarction (Figure 1). Computed tomography (CT) angiography of the aorta was normal. Blood sugar, lipid profile, electrocardiogram, and transthoracic echocardiogram were also normal. He was thrombolysed with an IV recombinant tissue plasminogen activator (rtPA) 0.9 mg/kg body weight dose at 428 minutes from symptom onset. Ten percent was given as a bolus dose and the rest through an IV infusion over one hour. Twenty-four hours after treatment, the patient had a flicker of movement in the right foot and grade two power in the left hip, knee, and ankle joints (NIHSS 7), suggesting that thrombolysis might have been effective. Aspirin and atorvastatin were started for secondary stroke prevention. Three months later, he could walk with a cane, and his joint position sense had improved, but he had urinary symptoms that required intermittent self-catheterization.

## Discussion

Our patient had acute-onset paraplegia with a sensory level at the L1 level. The diagnosis was dorsal myelopathy due to a spinal cord infarction. The MRI spine in the hyperacute phase may be normal, but it can rule out alternative causes like spinal cord compression, intramedullary hematoma, transverse myelitis, or neoplasm. The presence of a vertebral body infarction is a clue to a spinal cord infarct. MRI sagittal T2W sequence may show linear "pencil-like" hyperintensity and, on axial scans, an "owl eye" appearance. These MRI findings are not specific to spinal cord injuries [5]. Zalewski et al. proposed a diagnostic criterion for spinal cord infarction [2]. CT angiography should be done to assess the aorta for the etiology of the infarction. Aortic dissection is a contraindication for thrombolysis [6]. We treated our patient with IV rtPA at seven hours of symptom onset. We faced a therapeutic dilemma as IV thrombolysis in spinal cord infarction is not well established, and there is no known precedence of IV thrombolysis at seven hours. Our patient had paraplegia; this is a poor prognostic factor for spontaneous motor recovery, and this prompted us to discuss the option of thrombolytic therapy. The family understood this unusual situation and provided consent for off-label treatment. Spontaneous spinal cord infarcts progress more slowly than cerebral infarcts, perhaps due to the extensive collateral blood supply to the spinal cord through anterior medullary segmental arteries [1]. Stroke mechanisms for spontaneous spinal cord infarction are varied; this may be secondary to aortic diseases like atherosclerosis, dissection, or aneurysm, vertebral artery dissection, vasculitis, cardioembolic, hypercoagulable state, severe systemic hypotension, and fibrocartilaginous embolism [2].

The literature review showed only 19 instances of IV thrombolysis in acute spinal cord infarction (Table 1). One patient was thrombolysed twice. Seven out of 20 (35%) cases were initially thought to have cerebral infarcts, perhaps due to hemibody motor or sensory symptoms. In such a scenario, the absence of cranial nerve abnormalities and normal brain imaging should raise the suspicion of spinal cord infarction. Seventeen (85%) of the 20 patients were treated within 270 minutes and three beyond 270 minutes (at 285, 330, and 428 minutes, respectively). None of the patients had post-thrombolysis worsening due to hemorrhage. Three-month outcomes were available for 16 patients. Seven (44%) of the 16 patients had a good recovery (mRS 0-2). The outcome in the delayed time window (beyond 270 minutes) was good in two out of three patients (mRS 1 at 11 months, mRS 2 at three months, and mRS 3 at three months in our patient [6-19].

**Table 1:** Case reports of acute spinal cord infarction treated with IV thrombolysis NA: not available, MRI: magnetic resonance imaging, CT: computed tomography, HTN: hypertension, DM: diabetes mellitus, HCC: hepatocellular carcinoma, Rt: right, Lt: left, VA: vertebral artery, NIHSS: National Institute of Health Stroke Scale, ASA: anterior spinal artery syndrome, mRS: modified Rankin scale, SL: sensory level, IV: intravenous

Series	Age in years/sex	Risk factor/s	Clinical presentation	Stroke severity (NIHSS)	Imaging extent of lesion	Time to thrombolysis (minutes)	Stroke mechanism	mRS at 3 months
Current case	Middle-aged man	HTN	Paraplegia L1 SL	9	D10-L1	428	Idiopathic	3
Focke et al. [8]	17/F	None	Paraplegia L4SL	NA	Conusmedullaris	270	Idiopathic	0
Almutlaq et al. [9]	81/F	HTN dyslipidemia	Paraplegia D6 SL		C5-T4	285	Atherosclerosis	2
Jankovic et al. [6]	57/F	Dyslipidemia	Lt leg sensory-motor deficit	4	T2 upper level	135	Idiopathic	3
	83/M	Smoking, dyslipidemia	Paraplegia	10	T8 upper level	240	Atherosclerosis	5
	82/F	HTN, DM, dyslipidemia, smoking	Lt hemimotor sensory syndrome	9	C2	245	Atherosclerosis	3
	74/F	Smoking, dyslipidemia	Rt arm loss of dexterity, ataxia	11	High cervical	190	Atherosclerosis	1
Muller et al. [10]	68/M	Smoking, HTN	Quadriparesis, C4 SL	NA	Normal MRI	270	Idiopathic	3
Restrepo et al. [11]	71/M	Severe systemic atherosclerosis	Paraparesis	6	CT brain normal/ CT spine normal	110	Postprocedural	0
Lee et al. [12]	58/M	HTN, HCC, Rt VA embolisation for C2 metastasis	Rt hemiplegia, left sensory loss	NA	Rt anterolateral cervical cord	90 (25 mg IV and 10 mg intra-arterial)	Postprocedural	1
Koch et al. [13]	81/M	HTN	Paraplegia	NA	T10	220	Atherosclerosis	3
Etgen et al. [14] (repeat stroke on day 3)	72/F	Uncertain	Rt hemiparesis, ataxia	NA	CT brain normal	30	NA	NA
	72/F	Uncertain	Rt sensory motor syndrome	NA	C4 posterolateral cord	180	NA	NA
Dorodnicov et al. [7]	55/M	HTN, obesity, dyslipidemia	Quadriparesis	NA	CT brain normal, post lysis MRI C4 lesion	240	Atherosclerosis	NA
Wiszniewska et al. [15]	61/M	None	Rt hemiparesis	8	CT brain normal, MRI C6, right side infarct	90	Cervical canal stenosis, Osteophyte compressing vessel	1
Pikija et al. [16]	57/F	NA	ASA syndrome	NA	Cervical level	Within 270	NA	5
	57/M	NA	Anterior and posterior cervical cord syndrome	NA	MRI cervical spine normal	Within 270	Atherosclerosis	3
Lawson et al. [17]	58/M	Smoking HTN prior stroke	Rt arm and Lt leg weakness	NA	Normal MRI brain day 5, MRI: C3 Rt ventral cord	Within 270 minutes	NA	2
Oliveira et al. [18]	M/45	Smoking HTN dyslipidemia	Paraparesis	4	T6-10	330, tenecteplase 0.25mg/kg	Fibrocartilaginous embolism	1 at 11 months
Xiao et al. [19]	M/61	Smoking	Left hemimotor weakness	NA	CT brain normal post lysis, MRI: C2 anterolateral	120	NA	3

## Conclusions

In the setting of acute nontraumatic myelopathy, clinicians should consider the possibility of spinal cord infarction; an MRI scan should be done to rule out alternative causes. IV thrombolysis for spinal cord infarction seems reasonable, but there is no good-quality evidence to support the hypothesis. A randomized control trial is required to establish the efficacy and safety of thrombolysis in acute spinal cord infarction within four hours and thirty minutes and also beyond this time interval.
